# A Link Between Allergy and Hematological Malignancies? Focus on Possible Mechanisms and the Potential Role of Biological Therapies

**DOI:** 10.1002/clt2.70146

**Published:** 2026-03-11

**Authors:** Stefania Isola, Luca Gammeri, Federica Nuccio, Alessandro Allegra, Giorgio Walter Canonica, Sebastiano Gangemi

**Affiliations:** ^1^ Department of Clinical and Experimental Medicine School and Operative Unit of Allergy and Clinical Immunology Policlinico “G. Martino” University of Messina Messina Italy; ^2^ Department of Biomedical and Dental Science and Morphofunctional Imaging University of Messina Messina Italy; ^3^ Division of Hematology Department of Human Pathology in Adulthood and Childhood “Gaetano Barresi” University of Messina Messina Italy; ^4^ Department of Biomedical Sciences Humanitas University Pieve Emanuele Milano Italy; ^5^ Personalized Medicine Asthma and Allergy IRCCS Humanitas Research Hospital Milan Italy

**Keywords:** alarmins, allergy, hematological malignancies, IgE, immune dysregulation, innate lymphoid cells, monoclonal antibody, Th2 response

## Abstract

Immune dysregulation has been widely recognized in the international literature as an underlying condition for hematological malignancies and allergic disorders. This commonality has led researchers to study the potential association, positive or negative, between blood cancers and allergy, but the results remain unclear. The cellular and molecular mechanisms underlying allergic inflammation appear to have dual effects on immune surveillance, potentially positively or negatively influencing carcinogenesis in solid tumors. The same mechanisms may also play a role in the genesis of hematological malignancies, but there is little evidence in the literature to support this. In our review, we explored the possible link between the immune pathways involved in allergic responses and the mechanisms underlying hematological malignancies, focusing on Th2 responses, the activity of inflammatory cells, cytokines, and the emerging role of alarmins. Furthermore, our review aims to assess the association between biologics and the risk of neoplastic disease, with a focus on hematological malignancies. A deeper understanding of shared immune dysregulation pathways and the interactions between various cell types could lead to new preventive or therapeutic approaches for patients with hematological malignancies. Understanding the complex roles of various cellular and molecular mediators of Th2 inflammation in stimulating or inhibiting tumor growth could be a key goal of future research, paving the way for innovative targeted therapies, especially at a time when immunotherapy and monoclonal antibody therapies are increasingly important and effective.

## 1 Introduction

The European Academy of Allergy and Clinical Immunology established a working group for AllergoOncology to evaluate the relationships between cancer and allergy, study allergic problems in clinical oncology, and explore immunomodulatory mechanisms [1, 2].

The immune system is involved both in cancers and allergic diseases [3]. The worldwide increased prevalence of atopic disorders over recent decades led researchers to state that atopy influences other aspects of health besides the allergic responses, such as cancer, with a promoting or protective role. Allergic disorders, including asthma, allergic rhinitis, food allergies, and eczema, are common health problems that result from inappropriate immune system response to environmental antigens and are innocuous for most of the population. Atopy is a hypersensitivity immunological reaction that involves the activation and effects of the immune system cells (Th2, Th17, eosinophils, neutrophils, basophils, mast cells, and others) and many soluble mediators (IgE, IgG, cytokines and chemokines). In allergic diseases, there is an imbalance in the T‐helper 1 (Th1) and T‐helper 2 (Th2) pathways that is responsible for multiple immunological disorders such as allergies [4].

This review aims to examine the interplay between hematological cancer and allergies by exploring the involvement of immune pathways in allergic diseases and cancer development. The study seeks to investigate how cells and other mediators responsible for allergic diseases (e.g., IgE, Th2 cells, mast cells, eosinophils, innate lymphoid cells, alarmins) may have a role in hematological malignancies and mediate both tumor‐promoting and tumor‐suppressing effects in different contexts. We also analyze mechanisms that could be targeted to prevent and treat these conditions by focusing on immune pathways. Considering the increasing role and importance of biological therapies in treating various allergic disorders, we also investigated how these drugs can affect the development of hematological diseases.

## 2 Overview of the Current Hypotheses Explaining the Allergy‐Cancer Relationship

Over the years, various hypotheses and theories have been developed to try to explain a possible link between allergies and cancer. To better understand how the two conditions may be linked, it is necessary to understand how certain conditions can affect the immune system.

The “*hygiene hypothesis*,” defined initially by Strachan in 1989, explains the increase in allergic diseases in industrialized Western countries over the past century [5]. This theory proposes that reducing exposure to infections early in life for improved hygienic conditions modulates the immune system and promotes the development of chronic inflammatory diseases, such as allergies and autoimmune diseases.

Indeed, infections exert their effects by altering the Th1/Th2 balance, and thus, they protect against atopy and asthma by inducing a Th1 regulatory response. This “*hygiene hypothesis*” concept applies to a broader range of chronic inflammatory diseases in addition to atopy, such as cancer [6, 7].

The study of allergic disease is of considerable interest in hematopoietic malignancies, as both conditions affect the immune system.

Some proposed hypotheses explain the carcinogenic role of allergy and Th2 inflammation. One of these hypotheses is the antigenic stimulation hypothesis. This hypothesis proposes a relationship between atopy and an increased risk of cancer incidence due to the prolonged stimulation of the immunological system by chronic inflammation that drives oxidative damage and subsequent gene mutation. Another hypothesis, the Th2 skewing hypothesis, suggests that atopy drives an inappropriate skewing toward Th2 responses over potentially tumor‐eradicating Th1 immune responses, thereby creating an immunosuppressive environment that is permissive to cancer development at sites of atopic inflammation [8, 9, 10, 11]. Inflammation driven by allergic responses, particularly in chronic conditions such as asthma and atopic dermatitis, increases cancer risk at sites of allergic response [12, 13]. However, in turn, the increased immunosurveillance and prophylaxis afforded by allergy and its symptoms protect against cancer at sites distant from that of the allergic disease's primary manifestation [9]. To demonstrate this, two further hypotheses have been formulated. First, the immunosurveillance hypothesis, which suggests that a hyperstimulated immune system in allergic patients might detect and eradicate dysregulated cells, thus acting protectively against cancer. The prophylaxis hypothesis suggests that in some cases, the physical effects of allergic reactions in specific tissue may clear mutagenic triggers before malignant transformation can occur.

The exact nature of these relationships and their effects on cancer development remain debated, and contradicting evidence has rendered it difficult to conclude and likely reflects a combination of all four of the above‐proposed hypotheses [14]. The risk of developing a neoplasm may depend on the type of allergic condition, the severity of the disease, its duration, the therapy, and also the type of neoplasm [15, 16].

Numerous epidemiological studies have investigated potential associations between a history of allergies and the risk of developing onco‐hematological diseases, with conflicting results. These results reflect a state of enhanced immune surveillance and anti‐tumor defense among patients with allergic disorders and, on the other hand, a state of chronic immune system stimulation associated with increased cancer risk [17, 18].

Cooper et al. [19] have investigated the relationship between immune‐related conditions and adult acute lymphoid leukemia (ALL) in 811 patients with acute leukemias and 637 healthy controls. Their results support either a protective effect of enhanced immune surveillance or a harmful effect from antigenic stimulation concerning the risk for acute leukemia in adults.

In a population‐based cohort study of postmenopausal women, no association was found between allergic diseases and risk of myeloid or lymphoid malignancies. However, allergic asthma was associated with myelodysplastic syndrome [20].

A 2010 meta‐analysis reported significant inverse associations between a history of allergy, eczema, hay fever, and hives and childhood acute lymphoblastic leukemia but not acute myeloid leukemia (AML). Inverse associations were observed for acute lymphoblastic leukemia and asthma (odds ratio (OR) = 0.79, 95% CI: 0.61, 1.02), eczema (OR = 0.74, 95% CI: 0.58, 0.96), and hay fever (OR = 0.55, 95% CI: 0.46, 0.66) examined separately [21].

On the other hand, some studies provide new data on the correlation between allergies and the development of tumors, stating that in subjects suffering from allergies or other autoimmune diseases, the risk of developing hematological diseases such as lymphomas or leukemias would be more significant.

A study analyzing the relationship between the immune system and cancer, examining 66,000 patients between the ages of 50 and 76 who were under observation for eight years, indicated that hematological malignant diseases are more frequent in subjects with pollen allergy [22].

Soderberg et al. [23] investigate potential associations between several autoimmune diseases and hematological malignancies in 39,908 patients from the Swedish Cancer Registry and 149,344 population‐based controls. Psoriasis was positively associated with leukemia and non‐Hodgkin lymphoma (NHL). Sjögren's syndrome increased the risk of all hematological malignancies. An increased risk of several hematological malignancies was present in autoimmune hemolytic anemia and idiopathic thrombocytopenic purpura but not in asthma, suggesting chronic autoimmunity and immune stimulation as mechanisms contributing to the development of hematological malignancies.

Another study underlined this concept by evaluating the association among certain allergic disorders (asthma and hay fever), atopy upon skin‐prick testing, and specific cancers (breast, prostate, colorectal, lung, melanoma, and hematological cancer). No association was found between colorectal, breast and lung cancer, while there was a positive association between respiratory allergy and leukemia [24].

Several preclinical studies have been conducted to better understand the crosstalk between B‐cells‐related dyscrasia and allergic responses in patients with Multiple Myeloma (MM). There is no evidence of a clear correlation between allergies and MM [25, 26].

D'Arcy et al. [27] studied an elderly American population aged 66–99 with the first cancer diagnosed in a range between 1992 and 2013 (1,744,575 cancer cases vs. 100,000 cancer‐free control) to demonstrate if some allergic conditions, with a particular focus on rhinitis, asthma and atopic dermatitis (eczema) could reduce the risk of developing a cancer by promoting immune surveillance. Various types of cancer involving different organs were investigated, and hematological malignancies were among these. As previously demonstrated, allergic rhinitis has been shown to have an inverse association with 16 different types of cancers and a positive association with follicular NHL, prostate, and thyroid cancers. Allergic rhinitis was not significantly associated with the risk of either brain cancers or MM. Asthma was investigated after excluding those with a COPD diagnosis and was found not to be significantly associated with the risk of most cancer types, including MM. Eczema was significantly associated with HL and NHL, and there was a lower significant association with MM [27]. More recently, a meta‐analysis including 6 of 29 studies carried out by Liang et al. [28] confirmed that eczema significantly increases the risk of MM more in American studies than in European ones, suggesting that race, as well as environmental factors, may have an influence. It remains unclear whether allergic conditions can positively or negatively influence the development of MM. The limits reported in various case‐control studies suggest the need to investigate whether immunological backgrounds can be involved.

## 3 The Possible Involvement of Immune Cells

### 3.1 Th1/Th2 Imbalance

There are several potential mechanisms by which allergic conditions may contribute to the risk of hematopoietic cancers. These include features of allergic responses that may mediate enhanced anti‐tumor immune responses and allergy‐associated changes in immune responses that inhibit immune cell activation, resulting in less exposure to DNA mutations and potentially oncogenic activity. One key mechanism in this relationship is the imbalance between Th1 and Th2 cells that occurs in allergic individuals. Th1 cells play a key role in eliminating tumor cells by producing cytokines such as interferon‐gamma (IFN‐γ) that mediate anti‐tumor activity through macrophage activation, enhanced antigen presentation, and inhibition of neoangiogenesis [29]. Infiltration of the tumor microenvironment by cells of the Th1 cell system, including M1 macrophages, natural killer cells, and CD8+ T cells, is associated with improved cancer prognosis. In contrast, the role of Th2 cells in cancer is more controversial. The activity of Th2 cells, which are prominent in allergic responses and are characterized by elevated levels of IL‐4, IL‐5, and IL‐13, may support an inflammatory environment that promotes cancer progression by stimulating cell proliferation and limiting apoptosis [30]. Indeed, infiltration of the tumor microenvironment by Th2 cells, M2 macrophages, and type 2 innate lymphoid cells (ILC2), as well as immunosuppressive myeloid suppressor cells and regulatory T cells, is associated with a poorer tumor prognosis [31]. Indeed, several studies have shown that in several common tumor types, Th2 cells and associated cytokines contribute to tumor progression [32, 33, 34, 35, 36].

Similar results have been observed in the study of onco‐hematological diseases. Zhang et al. [37] reported dysregulation of Th1 and Th2 cell function, with a reduction in the number of Th1 cells and an increase in the number of Th2 cells in children with ALL.

Mori et al. [37] reported that the Th1/Th2 balance was biased toward Th2 in untreated diffuse large B‐cell lymphoma and that Th1/Th2 imbalance may play a role in lymphomagenesis.

In some lymphoid malignancies, increased Th2 cytokines correlate with disease progression. In cutaneous T‐cell lymphoma (CTCL), a dominant Th2 profile has been proposed to be associated with disease progression, and IL‐4 has important implications in this process [38].

MM has been reported to accompany various T‐cell abnormalities, including quantitative and functional defects of CD4+ and CD8+ T cells. Many authors have reported that Th1‐type immunity was found preferentially in cases of indolent disease and Th2‐type response predominated in cases of advanced MM and suggested that Th1 cells were suppressed in MM patients [39, 40]. However, other authors have shown that Th2 cells and cytokines may also play a protective role.

Surprisingly, an in vitro study demonstrated that IL‐4 overexpression significantly inhibited the growth of human and murine melanoma cells via p21‐mediated activation of the STAT6 pathway while also inducing increased expression of apoptotic cell death proteins [41]. Concerning hematological neoplasms, many Th2 cells in Hodgkin lymphoma (HL) are associated with better survival [42].

Highlights:

— Th1 cells play a protective role, infiltrating the tumor microenvironment and mediating the anti‐tumor immune response through the action of cytokines such as IFN‐γ.
— Th1/Th2 imbalance towards Th2‐related immune responses may play a crucial role in the progression and prognosis of hematological malignancies like ALL, untreated diffuse large B‐cell lymphoma, CTCL, and MM by creating an inflammatory environment that favors tumor cell proliferation.
— Th2 cells, as well as M2 macrophages and regulatory T cells contribute to tumor progression through the action of cytokines such as IL‐4.
— An imbalance towards Th2 cytokines has been observed in the context of several hematological malignancies, such as B‐cell lymphoma, CTCL and advanced MM.

### 3.2 Mast Cells

Granulocytes, including basophils, eosinophils, neutrophils, and mast cells, are derived from the myeloid lineage and perform important, often disparate, roles across the allergic disease spectrum. Although these cells and their mediators are commonly associated with allergic inflammation, they also exhibit several functions in malignancies, either promoting or restricting tumor growth.

A position paper from the EAACI AllergoOncology group highlighted epidemiological associations between granulocytes and mast cells, as well as responses to cancer treatments. Indeed, enhancing their activity could induce the secretion of mediators with antitumor properties [2].

The mast cell is a tissue‐resident inflammatory cell of bone marrow origin that responds to danger signals, participating in both innate and acquired immunity by immediately and delayed releasing inflammatory mediators. In fact, mast cells (MCs) are highly abundant at tissue sites that interface with the external environment, strategically located near blood vessels and sensory nerves. This localization enables them to rapidly sense and respond to a wide range of microenvironmental stimuli, a capacity supported by the diverse repertoire of receptors expressed on their surface. Upon activation through receptor‐specific ligand binding, MCs release mediators that are either preformed and stored within cytoplasmic granules or newly synthesized, thereby leading to different phenotypes and functional outcomes [43].

MCs, activated through IgE antibodies via high‐affinity FcεRI receptors expressed, are known to be pivotal in the pathophysiology of anaphylaxis and other immediate hypersensitivity reactions [43], but they may also contribute to hematologic cancer pathogenesis in different ways.

A pathologic excess of MCs leads to a pathologic condition called mastocytosis. This disease varies from benign or indolent forms to mastocytosis associated with bone marrow pathology, including myelodysplasia. It is usually diagnosed on the basis of characteristic skin findings, an elevated serum level of tryptase, and specific bone marrow findings.

In this section, the term tumor‐associated mast cells (TAMCs) is used to denote MC that are recruited to or activated in the tumor microenvironment (TME) by tumor‐derived chemokines, damage‐associated molecular patterns (DAMPs) and local alarmins and, along with other immune cells, become part of it. The generic term MCs is retained for MCs outside the tumor context or when discussing basic MC biology.

Their migration is principally mediated by stem cell factor (SCF) interaction with the c‐kit receptor expressed on the surface and supports their survival and accumulation in tumors. Additional mediators including bioactive lipids (e.g., S1P, LPI, LPA, LTB, PGE2) and alarmins (HMGB1 via TLR4; IL33 via ST‐2) interact with their respective receptors, resulting in cascade intracellular signals and consequent TAMCs' activation and degranulation [44].

TAMCs exert a dual effect on tumor development depending on their tissue localization and tumor type. The presence of TAMCs, as demonstrated in common malignant human tumors and animal models of tumor development, correlates with different prognoses varying on their localization (peritumoral/intratumoral) and the type and tumor stages. More specifically, peritumoral dislocation is associated with worse prognosis, while intratumoral MCs can have either good or bad outcomes depending on other undefined mechanisms [44]. In a few cases, they appear as non‐contributing bystanders [45].

In hematological cancers such as HL [46, 47, 48], NHL [49, 50, 51] and plasmocytoma [52, 53] MC infiltration correlates with poor prognosis. Conversely, in some solid tumors like breast cancer, MCs may have an anti‐tumorigenic role. In melanomas, their function is variable and depends on their microlocalization and the subtype of tumor [54, 55]. In others, they can have both pro and anti‐tumorigenic functions [45].

Concerning their function in tumor progression, histamine and other mediators released by activated TAMCs can suppress tumor growth but also, paradoxically, promote angiogenesis, a critical process for tumor survival and expansion. The exact impact of these factors varies depending on the type of malignancy and the tissue environment, but their involvement is evident in the pathophysiology of both allergic diseases and cancers, making them a point of interest in understanding these interactions [42].

Tryptases and chymases, specific serine proteases that can be pre‐formed or stored in secretory granules of MCs, have shown to have both direct and indirect influence on tumor biology, being central to angiogenesis and extracellular matrix remodeling. Compared to chymases, tryptase plays a more significant role in this process [56].

In particular, tryptases seem to have a crucial role in the initial phase of the angiogenetic process, as demonstrated by their action on various substrates that act on different levels of angiogenesis and the location of tryptase‐positive MCs near the blood vessel.

About 30 years ago, Blair et al. [57] demonstrated in vitro a direct action of tryptases on endothelial cells, obtaining an increase in capillary‐like structures, and thus proliferation, of human dermal microvascular endothelial cells (HDMCECs) incubated in human lung tryptase for 16 h.

More recently, literature has focused on the possible molecular mechanisms that are the basis of its pro‐angiogenic action.

Tryptases stimulate in vitro fibroblast proliferation and neovascularization through their proteolytic activity on protease‐activated receptor‐2 (PAR‐2), which in turn regulates the expression of urokinase plasminogen activator (uPA) and plasminogen activator inhibitor‐1 (PAI‐1), and via Angiopoietin‐2 (Ang‐2)/Tie‐2 [55].

Furthermore, tryptases indirectly contribute to extracellular matrix turnover by stimulating and/or altering its components. They activate metalloproteinases (MMP), leading to fibronectin degradation, stimulating procollagen synthesis, and releasing interleukins and angiogenic factors of the matrix, such as vascular endothelial growth factor (VEGF) and fibroblast growth factor‐2 (FGF‐2) [56].

Extreme conditions characterizing tumor microenvironment, in particular hypoxia, reactive oxygen species (ROS) accumulation and adenosine presence, and other pro‐inflammatory mediators, can influence MC polarization towards the pro‐tumoral phenotype (MC2 subtype). MC2s secrete ROS, proteases, a multitude of angiogenic mediators and growth factors, including histamine, angiopoietin‐1, VEGF, FGF‐2, tumor growth factor‐beta (TGF‐b), tumor necrosis factor‐alpha (TNF‐a), and IL‐18 and IL‐13 thus contributing, in numerous ways, to tissue remodeling and tumor development and progression [58].

Notably, MCs also contribute to lymphangiogenesis through the production of pro‐angiogenic (VEGF‐A, VEGF‐B, and FGF‐2) and pro‐fibrotic factors.

Since lymphangiogenesis is crucial for tumor metastasis formation and MCs were found in metastatic lymph nodes of cancer patients, MCs may have a role also in metastatic tumors. In addition, it has been demonstrated that they can induce epithelial‐to‐mesenchymal transition (EMT) through production of CXCL8/IL‐8 [59], facilitating metastatic behavior and chemotherapy drug resistance [60, 61].

Furthermore, TAMCs explicate immunosuppressive properties via the release of anti‐inflammatory cytokines, including interleukin‐10 (IL‐10), TGFb, and adenosine. These mediators stimulate histamine secretion from MCs, leading to vasodilation, increased capillary permeability, and Th1/Th2 inhibition through H2 receptors activation [58].

This immunosuppressive milieu supports tumor immune evasion.

A pathologic excess of MCs leads to a pathologic condition called systemic mastocytosis (SM). This disorder has variable clinical presentation ranging from indolent forms (ISM) to those associated with bone marrow pathology. It is usually diagnosed on the basis of characteristic skin findings, elevated serum level of tryptase, and specific bone marrow findings.

Individuals with SM may develop various hematologic abnormalities, including cytopenia, myeloproliferative or myelodysplastic syndromes, lymphoproliferative syndromes, and primary or secondary leukemias [62].

Systemic mastocytosis with associated hematological non‐mast cell disease (SM‐AHNMD) represents the second most frequent subtype of advanced systemic mastocytosis after ISM characterized by the coexistence of abnormal clonal MC proliferation and other non mast‐cell clonal hematological disorder, represented in over 90% of cases by a myeloid neoplasm (myelodysplastic syndromes, myeloproliferative neoplasms) and 10% by lymphoid or plasma cell ones. Patients with SM‐AHNMD demonstrated to have more frequent constitutional symptoms, worse prognosis, major risk of drug resistance and inferior overall survival compared with pure SM patients. Whether this pathogenetic correlation between MCs and non‐MC clones is based on a shared progenitor alteration or paracrine interaction within TME is still debated, but it further highlights how MCs influence and intersect hematological malignancies beyond the classical Ig‐E mediated pathways [63, 64].

Highlights:

— The role of mastcells in the pathogenesis of onco‐hematological diseases in bimodal and is influenced by tumor microenvironment.
— Peri‐tumoral localization of tumor‐associated mast cells (TAMCs) is usually associated with poor prognosis, while intratumoral infiltration has a variable course.
— MCs, by releasing pro‐angiogenic and pro‐fibrotic factors, can promote extracellular matrix remodeling, angiogenesis and metastasis processes.
— TAMCs exert immunosuppressive action through the release of anti‐inflammatory cytokines.
— Tryptase could be implicated in the process of neoangiogenesis, regulating fibroblast proliferation through proteolysis on the PAR‐2 receptor.
— The coexistence of systemic mastocytosis with a non‐mast cell hematological malignancy (SM‐AHNMD) further highlights how MC may contribute to the TME not only with their aberrant immune response but influencing the growth, survival and prognosis of associated hematological malignancy.

### 3.3 Eosinophils

Eosinophilia is observed in patients with various inflammatory and allergic conditions, as well as in several hematological malignancies.

In the context of hematological malignancies, eosinophilia can be of the reactive or neoplastic type. In the first case, eosinophil increase is secondary to the production of eosinophilopoietic cytokines. In the second case, the increase in eosinophils is related to the proliferation of a malignant clone [65].

Myeloid malignancies variably accompanied by eosinophilia include chronic myeloid leukemia, other myeloproliferative neoplasms (MPN), distinct variants of acute myeloid leukemia (AML), rare forms of myelodysplastic syndromes (MDS), some MDS/MPN overlap disorders, and a subset of patients with (advanced) SM. HL may present with peripheral blood eosinophilia or, less commonly, tissue or marrow eosinophilia. Approximately 10% of adult T‐cell leukemia/lymphoma cases are associated with reactive, IL5‐mediated peripheral blood eosinophilia, and 2%–20% of patients with NHL (mostly of T‐cell origin) have elevated eosinophilia [66, 67].

Some patients develop eosinophilic dermatitis associated with hematologic malignancies (EDHM), characterized by various skin manifestations, including pruritic macular, vesicular‐papular, or vesicular rashes. Chronic lymphocytic leukemia (CLL) is the most common malignancy associated with EDHM. Initially thought to be a hypersensitivity reaction to insect bites, many affected individuals have been found not to have been exposed to insects [68].

Eosinophils exhibit a bimodal behavior in the context of cancer. These cells may have anti‐tumorigenic properties, producing, for example, TNF‐α, granzyme, cationic proteins, and IL‐18. The same cells may also have pro‐tumorigenic activities, producing pro‐angiogenic factors. It seems that in solid tumors, eosinophils play mainly an anti‐tumorigenic role, while in HL, eosinophilia is associated with poor prognosis [69]. However, there is no evidence in the literature about the precise mechanisms through which eosinophils may influence the progression of hematological tumors.

Highlights:

— Eosinophils can have a pro‐tumorogenic or anti‐tumorogenic role.
— The pro‐tumor effect is mediated by the production of agents that promote neoangiogenesis. TNF‐α, granzyme, cationic proteins, and IL‐18 mediate the anti‐tumor effect.
— The exact mechanisms by which eosinophils exert these effects are not yet known.

### 3.4 Innate Lymphoid Cells (ILCs)

Innate lymphoid cells (ILCs) are a family of lymphocyte‐like cells of the innate immune system that are regulated by signals from the tissue microenvironment.

They exhibit pleiotropic effects, driving seemingly paradoxical responses such as tissue repair and immunopathology toward allergens or promoting tumorigenesis [70, 71]. ILC can be divided into three subgroups: ILC Group 1, comprising NK cells and ILC1; Group 2, which includes ILC2 alone; and Group 3, containing Lymphoid Tissue inducers (LTi) and ILC3 cells. While Group 1 ILCs mainly exert anti‐tumor activity, Group 2 and Group 3 ILCs are protumorigenic in nature [72, 73, 74]. Many studies focus on identifying regulatory factors that determine the pro‐ or anti‐tumor role of ILCs, as well as the role of various mediators (such as hormones, vitamins, cytokines, or gas transmitters) in regulating the biology of ILCs within the context of tumor immunity in order to identify new therapeutic possibilities in cancer patients. Early evidence suggests that ILCs may play a role in tumor development by producing tumor‐promoting cytokines and contributing to an immunosuppressive environment. Observations to date indicate a complex network of interactions between ILCs and the bone marrow microenvironment. Through their diverse functions, ILCs may be involved in early‐stage disease progression and play a protective role against carcinogenesis.

A key challenge in cancer immunotherapy is inhibiting the multiple immunosuppressive circuits in the TME and fostering immunomodulatory processes to promote tumor eradication. In this context, the innate immune system cells play a key role in dictating the polarity of the TME [75, 76, 77].

A growing body of preclinical and clinical data supports the role of ILCs in the pathogenesis of MM. Therefore, targeting ILCs may be of clinical benefit.

Recently, ILC1s affected the growth of AML cells in vivo. In vitro experiments revealed that ILC1s induced apoptosis of leukemia Stem Cells (LSC), whereas at low densities, ILC1s inhibited the differentiation of LSC to leukemic precursors. Both effects of ILC1s were mediated by IFNγ produced by these cells following cell‐to‐cell contact with LSCs.

Similarly, an increased population of NCR—ILC3s was also found in the peripheral blood of patients with CLL [78, 79].

In AML, at diagnosis, ILCs are significantly dysregulated in frequency, subtype composition, and function [80, 81]. Within the entire ILC family, NK cells play a particularly key role in cancer surveillance, exhibiting “spontaneous” cytotoxicity independent of costimulatory signals or gene rearrangement events [82, 83].

In patients with CLL, ILC counts are increased, and the function of type 1 ILCs is altered, similar to what has been previously demonstrated for NK cells [79].

The ILCs that mainly constitute the allergic subject are ILC2s. Their role in the pathogenesis of allergic diseases is well understood and continually updated. However, there is still no certainty on the correlation between ILC2s and cancer. The little evidence present in the literature is often conflicting. Trabanelli et al. [84] observed that ILC2s activated by neoplastic cells in models of promyelocytic leukemia induced monocytic myeloid‐derived suppressor cells, thereby promoting tumor growth. ILC2s would activate suppressor cells by secreting IL‐13.

Recently, Li et al. [85] published a study on the potential anti‐tumor effects of ILC2s. In vitro studies have demonstrated that granzyme B, produced by these cells, exerts a cytolytic effect on solid tumor cells and leukemia models.

A peculiar characteristic of these cells was observed when ILC2s were studied in patients with MM. MM‐ILC2, derived from peripheral blood and bone marrow, can adapt to the microenvironment, assuming, in different contexts, either anti‐tumor or pro‐tumor characteristics [86]. In samples derived from peripheral blood, DNAM‐1 expression prevails, and the granzyme mediates the cytolytic and anti‐tumor action of ILC2s. Therefore, MM cells were susceptible to killing by MM‐ILC2s derived from peripheral blood. In contrast, in bone marrow‐derived MM‐ILC2s, downregulation of DNAM‐1 and upregulation of TIGIT were observed, which mediates cell death in ILC2s upon recognition of ligands expressed by MM cells. TIGIT blockade also restored DNAM‐1 expression and the cytolytic and anti‐tumor activity of bone marrow‐derived MM‐ILC2s [86].

Highlights:

— ILC2s are a major player in the allergic inflammatory response. However, the mechanisms by which these cells can influence tumorigenesis or the anti‐tumor immune response are not well understood.
— In vitro studies have demonstrated the ability to activate monocytic myeloid‐derived suppressor cells through the production of IL‐13.
— Granzyme B produced by ILC2s would have a cytolytic effect on cellular models of leukemia.

## 4 The Role of Immune Proteins

### 4.1 IgE

Recently, there has been a rapid expansion of studies evaluating biological indicators of allergy history and immune functions, including levels of IgE, inflammatory‐related cytokine, and concentration of immune regulatory proteins, fundamental to overcoming the methodological limitations associated with prior studies evaluating self‐reported allergy history about cancer risk, and required to clarify associations observed [3, 87].

The possibility of a dependence between IgE‐mediated allergies and the prevalence of cancer is an interesting research topic, and it is known that various types of cancer can have different associations with atopic diseases.

In 2008, the AllergoOncology working group released a position paper on the potential utility of IgE in counteracting tumor cell activity. IgE could play a role in natural tumor surveillance and could also be used for tumor control in the context of active and passive immunotherapy [88]. Later, the same working group suggested that although elevated serum IgE levels are generally associated with allergic conditions, very low or absent IgE levels could hinder cancer surveillance. Therefore, the working group suggested using ultralow IgE levels as a possible biomarker for cancer risk [89].

Early studies ascribe potential roles for IgE, allergy, and atopy in protecting against specific tumor types, with a corresponding increased cancer risk associated with IgE immunodeficiency [90].

In this context, elevated serum IgE associated with allergic diseases is believed to confer a protective role against malignancy. IgE causes the rapid expulsion of pathogens, natural toxins, and carcinogenic antigens before they can trigger malignant development. Hence, immunocompromised persons have a higher incidence of some malignancies.

Several IgE‐based active and passive immunotherapeutic approaches have been explored in various in vitro and in vivo models, suggesting the potential of IgE immunotherapies in oncology [91].

However, the immunosurveillance theory, invoked to explain the observed inverse association between allergy and cancer with the participation of elevated levels of IgE (whose protective role against carcinogenesis was confirmed by Van Hemelrijck) and the release of Th2 cytokines that recruit and activate eosinophils, macrophages, natural killer cells, and others, in atopy is now called into doubt by other observations [92].

In a cohort of patients affected by Mycosis fungoides, the most common expression of CTCL, serum IgE levels and eosinophil counts were higher in patients with advanced MF compared with patients with less advanced disease [93]. This effect, however, could be due to the ability of neoplastic T cells to secrete Th2 cytokines, such as IL‐4 and IL‐13. These cytokines, in turn, mediate the production of IgE and IL‐5 and promote reactive eosinophilia. Consequently, patients with more extensive skin involvement will have higher IgE and eosinophil levels [93].

### 4.2 The Emerging Role of Alarmins

Alarmins are endogenous, constitutively expressed, chemotactic, and immune‐activating proteins/peptides released due to degranulation, cell injury, or death, or in response to immune induction. Alarmins play important roles as initiators and participants in various physiological and pathophysiological processes, including host defense, regulation of gene expression, cellular homeostasis, wound healing, inflammation, allergy, autoimmunity, and oncogenesis [94]. Alarmins function as intercellular signals that defend by interacting with chemotactic and pattern recognition receptors (PRRs) to galvanize immune cells in host defense. Only when alarmins are released in excess by severe injuries and maximal stimulation do they result in the dangerous effects of the potentially lethal cytokine storm. Several studies have identified a role for epithelial‐derived cytokines, such as IL‐25, IL‐33, and thymic stromal lymphopoietin (TSLP), in the pathogenesis of allergic diseases, particularly asthma [95].

In the context of tumor immunity, alarmins appear to exhibit both beneficial and harmful effects. Many alarmins can promote anti‐tumor immunity by activating Antigen‐presenting cells (APCs), including dendritic cells and macrophages. However, some alarmins can also promote tumor progression by enhancing inflammation through the production of growth factors and angiogenesis. The nucleosome‐binding protein High‐mobility group box 1 (HMGN1) is a potent alarmin that binds TLR4 and induces antigen‐specific Th1 immune response. HMGB1 is also involved in the onset of numerous diseases, including some allergic diseases, such as asthma. This alarmin is implicated in the pathogenesis of the disease through several signaling pathways. The primary HMGB1 signaling pathways involved in asthma development are HMGB1/TLR4/NF‐κB and HMGB1/RAGE [96].

HMGN1 preferentially induces Th1‐polarized immune responses and has been shown to promote the development of protective anti‐tumor immunity [97, 98]. However, recent studies suggest that HMGB1 can also promote tumor progression, metastasis, and the formation of an immunosuppressive tumor microenvironment by promoting inflammation [99]. The functional relationship between HMGN1 and carcinogenesis, including leukemia, has been highlighted in recent studies [100]. HMGB1 has been demonstrated as one of the major players in several cancers, including colon, breast, lung, prostate, cervical, skin, kidney, stomach, pancreatic, liver, bone, and blood cancer [101]. HMGB1 has been confirmed to exert various effects on pathological symptoms and different stages of hematological malignancies and, may serve as a beneficial biomarker for diagnosing and prognosing hematological malignancies. For example, elevated expression of HMGB1 may be an important biomarker for the development and progression of T‐cell lymphoma [102]. Moreover, HMGB1 is related to the chemoresistance of various hematological malignancies.

HMGB1 is essential for reactive oxygen species (ROS)‐mediated tumor cell differentiation. Upregulation of this alarmin promotes autophagy and the degradation of PML‐RARα. In particular, HMGB1 regulates interactions between the ubiquitin‐binding adapter protein p62/SQSTM and PML‐RARα, promoting PML‐RARα degradation [103].

Recently, several studies have reported that dysregulation of the TSLP/TSLPR pathway may be responsible for the pathogenesis of solid and hematological tumors [104].

In many pediatric acute lymphocytic leukemia patients, mutations have been identified in genes encoding the TSLP signaling pathway components. In particular, these mutations affect patients with a clinical profile that overlaps with that of patients with the Philadelphia chromosome (Ph+) but without the typical translocation, defined as Ph‐like forms [105]. Approximately half of these patients exhibit chromosomal rearrangements that involve the gene encoding TSLPR. The determined alterations lead to an increased expression of TSLPR [106]. Furthermore, genetic alterations that lead to an increased expression of TSLPR are associated with an unfavorable form of the disease.

Vetter et al. [107] found increased expression of TSLPR in some patients with B‐cell precursor acute lymphoblastic leukemia. Stimulating cell cultures with TSLP, the authors observed that cells with higher surface expression of TSLPR exhibited increased proliferation and greater activation of the JAK/STAT signaling pathway. Using an anti‐TSLP reversed these effects, demonstrating the active role of TSLP in the proliferation of neoplastic cells and the therapeutic potential of a targeted therapy.

Interleukin 33 (IL‐33) is a cytokine belonging to the IL‐1 family, released following cellular damage and increased during inflammation. IL‐33 is among the alarmins and can stimulate several immune system effectors, regulating numerous immune responses [108]. Being therefore implicated in regulating the immune response, IL‐33 can also influence the immune system's anti‐tumor response. In particular, this alarmin may have a dual role in the pathogenesis and progression of tumors [109]. In myeloproliferative diseases, IL‐33 stimulates myelopoiesis by stimulating the production of growth factors in the bone marrow. Instead, in lymphoproliferative diseases, the same alarmin stimulates a pro‐inflammatory anti‐tumor response [109]. IL‐33 would promote the migration of pro‐inflammatory cells into the tumor microenvironment, promoting angiogenesis, proliferation, and metastasis. The anti‐tumor effect is promoted by recruiting NK cells and cytotoxic T cells, mediating the immune response against tumor cells [110].

**FIGURE 1:**
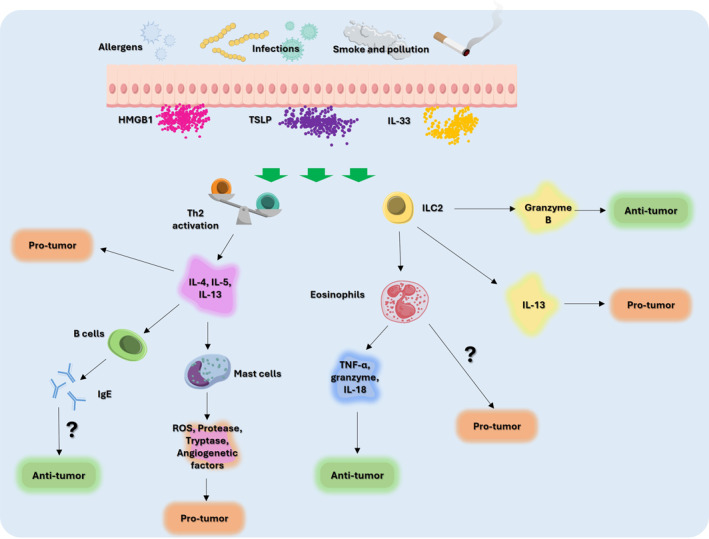
Representation of possible mechanisms linking mediators of the allergic response to their anti‐tumor and pro‐tumor effects in hematological malignancies. Question marks represent those pathways for which no clear evidence exists in the literature.

Figure 1 summarizes the mechanisms that link the protagonists of the Th2 inflammatory response to the pathogenesis of onco‐hematological processes.

Highlights:

— Alarmins can exert anti‐tumor activity by stimulating and activating APCs. Conversely, by promoting the inflammatory environment, some alarmins can stimulate angiogenesis and tumor growth.
— HMGB1 would exert an immunosuppressive action, promoting tumor progression in both solid and hematological forms.
— Mutations in genes involved in the TSLP/TSLPR signaling pathway have been identified in some leukemia cells. These may be responsible for the progression of certain hematological tumors and could serve as a future therapeutic target.
— IL‐33 promotes neoangiogenesis in the tumor microenvironment of myeloproliferative diseases, while it mediates cytotoxic and anti‐tumor action in lymphoproliferative processes.

## 5 The Role of Biological Therapies in Onco‐Hematological Processes

From the above, there is evidence in the literature regarding the protective role that immune system elements involved in allergic inflammation play towards hematological diseases. On the other hand, several sources claim that some of these cells and cytokines could favor the genesis and progression of neoplastic diseases. In light of the now widespread use of biological therapies aimed at blocking the action of one or more components of this system, it is essential to ask a particular question: can these therapies favor the development and progression of these pathologies, or do they play a protective role?

Biological therapies used to treat severe forms of certain allergic diseases, such as asthma and urticaria, utilize monoclonal antibodies (mAb) directed against specific elements of immuno‐phlogosis. In particular, these antibodies can antagonize specific cytokines, cellular receptors, alarmins, or even IgE. The literature includes several studies regarding the association between the use of mAb and the risk of hematological tumors.

The most apparent evidence to date concerns omalizumab, the first mAb used to treat allergic asthma. Omalizumab is a humanized, recombinant, anti‐IgE mAb that binds to free IgE at the FcεRI binding site, thereby preventing its interaction with the receptor present on cells of the immune system, primarily on MCs [111]. In this way, the immunological cascade is prevented. This antibody is used to treat allergic asthma, urticaria, nasal polyposis, and food allergies.

In 2012, Busse et al. [112] utilized data extracted from clinical trials conducted since the initietion of the drug registration. Out of 11,459 patients, only 25 developed malignancies (14 cases in patients treated with omalizumab and 11 among patients treated with placebo). This initial analysis found no correlations between the use of this mAb and the development of neoplasms.

A few years later, Long and his team confirmed the drug's safety [113], analyzing data from the EXCELS study, a prospective observational cohort study of patients with moderate to severe allergic asthma treated with omalizumab. However, a letter to the editor shortly thereafter highlighted the limitations of the EXCELS study, questioning whether the increased risk of cancer development could be ruled out with omalizumab therapy. The authors highlighted that there was selection bias in the study and that a high rate of study discontinuation may have limited the study's ability to exclude a risk of malignancy with treatment [114].

An analysis using VigiBase, the World Health Organization's global database of individual case safety reports, reported an increased incidence of cancer among patients receiving biological therapy. Of the 1380 neoplasm cases analyzed, only 21 developed a hematologic malignancy [115]. However, more recent studies and accurate data analyses from clinical trials and real‐world data have demonstrated the drug's complete safety [116]. Bagnasco et al. [117], in a more recent meta‐analysis published in the World Allergy Organization Journal, found no correlation between tumor development and the use of omalizumab.

Overall, current scientific evidence suggests that omalizumab may not have a direct or indirect role in tumor pathogenesis, especially in hematological tumors. Indeed, some authors have proposed using omalizumab to treat pruritus unresponsive to common therapy in patients with myeloproliferative neoplasms [118].

Antibodies directed against IL‐5 or its receptor are now widely used for treating inflammatory diseases in which Th2 inflammation prevails. This type of antibody is called anti‐eosinophil precisely because it acts on this type of granulocyte cell. Reslizumab is a humanized IgG4 kappa mAb that targets IL‐5 and thus blocks the maturation, activation, and survival of eosinophils. In 2020, Virchow et al. [119] studied the safety profile of this drug by analyzing data extracted from 6 clinical studies in asthmatic patients treated with reslizumab for over 12 months. There were no significant differences in the incidence of solid or hematological tumors between patients receiving therapy and those in the control group. Another anti‐IL5 mAb is mepolizumab. A first analysis in 2019 evaluated the overall safety profile for the first time over 12‐month period. Specifically, data from 347 patients extracted from the COLUMBA study, an open‐label long‐term extension safety study, were used. In a mean treatment period of 3.5 years, no patient developed onco‐hematological pathologies [120]. Data analysis from the COSMEX study also did not show an increased incidence of lymphoproliferative or myeloproliferative processes in patients treated with mepolizumab [121].

In 2018, the BORA Phase 3 extension trial evaluated this treatment's long‐term efficacy and safety [122]. The study included 1926 patients enrolled between 2014 and 2016. Of these, 1576 patients were treated with benralizumab. A total of 12 patients (1%) developed a malignancy during follow‐up; among these, four were hematological malignancies. However, among the 12 cases of malignancy, only 1 (prostate cancer) was treatment‐related, while all other cases were classified as therapy‐independent [122].

Dupilumab is a human mAb that targets the IL‐4 alpha receptor and treats atopic dermatitis, severe asthma, and nasal polyposis. The Phase 3 LIBERTY AD SOLO 1 and LIBERTY AD SOLO 2 studies in patients with atopic dermatitis showed no increase in the incidence of solid or hematologic tumors [123]. Heterogeneous and inconclusive data are reported in the literature regarding the associations between dupilumab use and the onset and progression of lymphomas.

Hasan et al. [124] reported an increase in CTCL cases in patients with atopic dermatitis who were treated with dupilumab. In the cohort of AD patients treated with dupilumab, the authors found an increased risk of developing CTCL (odds ratio 4.1003, 95% confidence interval 2.055–8.192) and that more than half of CTCL cases were diagnosed more than 1 year after dupilumab use. However, the authors themselves acknowledge that their study had limitations, including potential misclassification in the database and the failure to assess disease severity. According to the authors, there is not necessarily a causal relationship, and further studies should be conducted. In addition, prolonged dupilumab therapy has been linked to exacerbation of CTCL; however, given the brevity of treatment in the reported patient, it remains uncertain whether the drug contributed to the rapid progression of disease [125, 126]. Conversely, blockade of IL‐4/IL‐13 signaling through dupilumab has been shown to suppress malignant T‐cell proliferation and enhance antitumor immune responses in Sezary syndrome [127].

In recent months, a series of case reports has been published suggesting a possible link between dupilumab and the unmasking or progression of CTCL [128, 129, 130]. A recent literature review analyzed 35 studies comprising case reports, case series, and retrospective studies. The results in the literature are conflicting. In some studies, the drug alleviated symptoms, while in others, it could be a triggering mechanism. However, these studies had several limitations, including potential missed early diagnosis, small sample sizes, lack of standardized treatment duration, limited long‐term follow‐up, and possible persistent or atypical presentations initially diagnosed as AD [131]. Another recent review also demonstrated that the associations reported in the literature are more likely due to misdiagnosis of AD or to unmasking of pre‐existing CTCL [132].

Bettolini et al. [133] in a recent retrospective study of 139 patients with one or more hematological comorbidities treated with dupilumab, demonstrated that hematological disease remained stable in 82.7% of patients. The data available in the literature therefore do not justify not using a highly effective drug. Patients with atypical manifestations of moderate‐severe AD must be better studied in the case of suspected CTCL [132].

Larger studies and extended follow‐up are likely necessary to enable a more rigorous assessment of the drug's effects.

Finally, the latest addition to the treatment of severe asthma is tezepelumab, an anti‐TSLP antibody. As we have seen, the role of TSLP in the genesis of hematological tumors is somewhat ambiguous. It can behave differently depending on the type of tumor, exhibiting both anti‐tumor and pro‐tumor actions. However, several studies report how the use of anti‐TSLP mAb often negatively affects tumor growth, leading this antibody to be a potential future therapy for some types of hematological neoplasms.

Studies on tezepelumab have not found an increase in the incidence of tumors. Data extracted from a Phase 3 study in 1061 patients (529 treated vs. 532 controls) did not report differences in the incidence of tumors between the two groups (both 0.8%) [134]. Overlapping data were reported in the results of the Phase 2 CASCADE study [135]. Furthermore, recently, preclinical studies have demonstrated that HZ‐1127, a humanized anti‐TSLP, is capable of inhibiting TSLP‐dependent STAT5 activation. This antibody may be a promising candidate for treating certain types of cancer [104].

The safety of using biological drugs, in terms of the risk of developing hematological neoplasms, is also confirmed by data extracted from the FDA's Adverse Event Reporting System (FAERS) and the European database of reports of suspected adverse reactions to drugs.

Table 1 summarizes the characteristics and main results of safety studies of biological therapies used for the treatment of allergic or Th2‐mediated diseases.

**TABLE 1:** Clinical studies and literature with evidence on the incidence of hematological or solid tumors among patients treated with biologics.

Author/year	Target Ab	Type of study	N° and features of patients	Results
Busse et al. [112], 2012	Anti‐IgE (omalizumab)	Pooled analysis	11,459 patients in 67 Phase I to IV clinical trials (omalizumab‐treated, *n* = 7789; control, *n* = 4252)	Malignancies were identified in 25 patients, with an incidence rate per 1000 patient‐years of observation time for omalizumab‐ and placebo‐treated patients of 4.14 (95% CI, 2.26–6.94) and 4.45 (95% CI, 2.22–7.94), respectively, and a corresponding rate ratio of 0.93 (95% CI, 0.39–2.27). The analysis showed no association between omalizumab treatment and the risk of malignancy
Long et al. [113], 2014	Anti‐IgE (omalizumab)	Clinical trial (EXCELS: prospective observational cohort study)	7857 patients (omalizumab‐treated, *n* = 5007; control, *n* = 2829)	Malignancy rates were similar in the omalizumab and non‐omalizumab cohorts, with a rate ratio of 0.84 (95% CI, 0.62–1.13)
Li et al. [114], 2015	Anti‐IgE (omalizumab)	Comment	—	The authors highlight the limitations of the EXCELS study (unmeasured/uncontrolled confounding factors, selection bias and initial exclusion of patients with a history of cancer or premalignant conditions, high rates of study discontinuation) which do not allow to exclude a risk of malignancy with treatment with omalizumab
Mota D. et al. [115], 2021	Anti‐IgE (omalizumab)	Disproportional analysis (VigiBase)	515.120 neoplasms (2000–2020)	A total of 1380 neoplasm were associated with omalizumab (breast cancer *n* = 232; ROR [95% CI] = 4.12 [3.61–4.69]) and (lung cancer *n* = 85; ROR [95% CI] = 3.94 [2.45–3.76]). Omalizumab may be associated with a significantly higher risk of malignancies, but confirmatory studies are needed
Ali Z. et al. [116], 2022	Anti‐IgE (omalizumab)	Nationwide registry‐based cohort study	1444 (998 women, 446 men) exposed to omalizumab and unexposed; age‐ and sex‐matched. Mean follow‐up time was 3.4 years	A total of 266 cancers (97 men, 169 women) were observed: 21 (1.5%) among exposed and 245 (1.7%) among unexposed. No increased risk of cancer associated with omalizumab used for treatment of CU or asthma
Bagnasco D. et al. [117], 2022	Anti‐IgE (omalizumab)	Metanalysis	Over 40,000 patients affected by asthma, urticaria, rhinosinusitis treated with omalizumab	There is no documented increased carcinogenic risk in long term treated patients
Virchow J.C. et al. [119], 2020	Anti‐IL5 (reslizumab)	Pooled analysis	1758 from 5 randomized controlled trials and 1 open‐label extension study; 730 received placebo and 1028 reslizumab 3.9 mg/kg for more than 12 months.	AEs and serious AEs occurred in higher percentages of patients in the placebo group (81% and 9%) than in the reslizumab group (67% and 6%). No significant difference in the incidence of malignancies was seen when compared with placebo or the general population
Khatri S. et al. [120], 2019	Anti‐IL5 (mepolizumab)	Clinical trial (multicenter, open label, long‐term study)	347 patients treated for 3.5 years from DREAM study	326 (94%) reported at least 1 AE during treatment, of which 97 (28%) were drug‐related AEs. The most common AEs were respiratory tract infection, headache, asthma exacerbation, and bronchitis. Six (2%) patients reported malignancies with incidence rates similar to the general population. No cases of hematological malignancies were noted
Khurana S. et al. [121], 2019	Anti‐IL5 (mepolizumab)	Clinical trial (COSMEX: multicenter, open‐label, long‐term, Phase IIIb study)	340 patients (339 treated with mepolizumab for 2.2 years) from MENSA, SIRIUS and COSMOS studies	315 (93%) reported on‐treatment AEs of which 51 (15%) was related to treatment. The most common AE was asthma exacerbation (34 patients; 10%). Malignancies were reported in 8 (2%) patients: Basal cell carcinoma (*n* = 2), prostate cancer (*n* = 2), breast cancer (*n* = 1), colon adenocarcinoma (*n* = 1), melanoma (*n* = 1). No cases of hematological malignancies were observed
Busse W. et al. [122], 2019	Anti‐IL5Rα (benralizumab)	Clinical trial (BORA: randomized, double‐blind, parallel‐group, Phase III extension study)	1576 patients from SIROCCO/CALIMA, *n* = 783 benralizumab Q4W and *n* = 793 benralizumab Q8W up to 2 years	The adjudicated malignancy rate in BORA was low (12 of 1576 patients). One malignancy related to treatment (prostate cancer) occurred 3 days after the second dose. Insufficient evidence to support a causal relationship between benralizumab and cancer. No treatment‐related hematological malignancies were observed
Hasan I. et al. [124], 2024	Anti‐IL4Rα (dupilumab)	Retrospective cohort study	AD + dupilumab (cohort 1, *n*° = 22,888), AD + no dupilumab (cohort 2, *n*° = 22,871), AD + dupilumab + no DMARDs.	An increased risk of CTCL was found in cohort 1(OR 4.1003, 95%CI 2.055–8.192). The increased risk persisted after exclusion of prior disease‐modifying antirheumatic drug use (OR 3.202, 95%CI 1.573–6.514).
Ma et al. [128], 2025	Anti‐IL4Rα (dupilumab)	Retrospective cohort study	14,936 dupilumab‐treated patients, 734126 ICS/LABA‐treated patients	Dupilumab‐treated patients were found to have a higher risk of lymphoma (54 vs.*versus* 43 cases, hazard ratio (HR) 1.79, 95% CI 1.19–2.71)
Accetta et al. [129], 2025	Anti‐IL4Rα (dupilumab)	Case series	136 cases of CTCL, 18 had previously received dupilumab	The study does not demonstrate a direct correlation between the two events. The CTCL manifestation may have been exacerbated by treatment, or the early stage CTCL may have been mistaken for AD
Li et al. [130], 2025	Anti‐IL4Rα (dupilumab)	Case series	three cases (treated for AD)	The authors do not exclude that CTCLs were misdiagnosed as AD
Menzies‐Gow A. et al. [134], 2021	Anti‐TSLP (tezepelumab)	Clinical trial	1061 patients, *n* = 529: Tezepelumab 210 mg every 4 weeks; *n* = 532: Placeb for 52 weeks.	The incidence of cancer (0.8% in each group) did not differ between the trail groups

Abbreviations: AD, atopic dermatitis; ADs, adverse events; CTCL, cutaneous T‐cell lymphoma; CU, chronic urticaria.

## 6 Discussion

From our review of the literature to date, there is considerable evidence confirming a correlation between the immunological mechanisms underlying allergic diseases and those implicated in the genesis and progression of neoplastic diseases. The data we have gathered support our hypothesis that cells and cytokines involved in Th2 inflammation influenced the development of hematological neoplasms. However, the evidences and studies are not as robust as those available for solid tumors.

Although the topic remains unclear, the various pathogenetic hypotheses proposed by different authors appear reasonable. Allergic diseases unbalance the Th1/Th2 ratio to the detriment of those cells that would tipically perform an anti‐tumor action. Th1 cells are important in immunosurveillance because they produce IFN‐γ, a cytokine that exerts an anti‐tumor action [29]. Furthermore, the chronic inflammation that develops in subjects with allergies, such as respiratory allergies, determines the creation of an inflammatory microenvironment in which oxidative stress prevails, elements that favor carcinogenesis. The results derived from statistical analyses of the various case studies present in the literature often give conflicting results. Often, some authors have highlighted how different types of tumors can be more or less incidental in different atopic patients. For example, some studies have actually noted a greater association between allergic respiratory diseases and hematological tumors [22, 24, 28]. On the other hand, several studies have reported the absence of a direct correlation, hypothesizing that in allergic patients, a state of enhanced immunosurveillance could develop, responsible for greater protection against the development of tumors [25, 26, 27].

Among the various cells implicated in allergic inflammation are MCs, whose role in the development and progression of hematological neoplasms remains unclear. It is known that MCs can often be found in the tumor microenvironment, where they can contribute to the growth and spread of neoplastic cells through the production of various angiogenic and pro‐fibrotic factors [58]. The literature delves more deeply into the potential role of MCs in solid tumors; however, the mechanisms that correlate these cells with hematological neoplasms are not well understood. Despite this, it is known that subjects with mastocytosis have a greater tendency to develop hematological tumors [62]. It is appropriate to investigate the role of MCs further in the development of non‐solid tumors, given the evidence of a correlation. Eosinophils also appear to have a dual role in regulating the anti‐tumor response. On the one hand, eosinophils may protect against developing hematological neoplasms through the production and release of granzyme, TNF‐α, cationic proteins, and IL‐18. On the other hand, in a still unknown way, eosinophilia is associated with forms of tumors with poor prognosis, indicating a possible pro‐tumor activity [69].

In allergic subjects, the activity of ILC2s prevails, and they have an active role in the progression of tumor growth. The IL‐13 produced by these cells would favor the activity of myeloid‐derived suppressor cells in models of promyelocytic leukemia, favoring their proliferation [84]. Conversely, the same cells can favor tumor cytolysis by producing granzyme B [85]. However, studies on MM models have demonstrated this duality, highlighting how the pro‐ or anti‐tumor activity of ILC2s is influenced by the microenvironment in which they are found [86].

Today, the role of the so‐called alarmins in the pathogenesis of many diseases, not only allergic ones, is increasingly relevant. Indeed the literature indicates that alarmins, such as HMGB1, TSLP, and IL‐33, can influence the development of both solid and hematological tumors. HMGB1 has been correlated with numerous solid and hematological tumors, assuming a potential role as a diagnostic and prognostic biomarker. This alarmin is important in ROS‐dependent tumor proliferation processes [101, 102, 103].

Several studies have shown that TSLP can promote tumor proliferation, especially following a greater expression of its receptor on the surface of neoplastic cells [104, 106]. Instead, IL‐33 would exhibit a dual behavior, depending on the tumor type, with anti‐tumor activity in the context of lymphoproliferative processes and pro‐tumor activity in myeloproliferative processes [109].

Although the mechanisms linking allergic inflammation cells and mediators to the development and progression of hematological tumors are not yet clear, the evidence regarding the safety of biological drugs used to treat allergic diseases is now clear. Using mAbs, molecular and cellular pathways are blocked, which, in some contexts, would have a pro‐tumor role and, in others, an anti‐tumor role. For example, using omalizumab and blocking IgE should block a mechanism that is instead active against oncological diseases. However, today, we have data from many years of drug use, and no increase in the incidence of hematological neoplasms has been observed in the treated populations. The same could be said for therapies that block eosinophilic inflammation. Furthermore, although anti‐TSLP mAbs have been recently introduced, it has been demonstrated in in vitro models that these antibodies exert a potent anti‐tumor action [104]. This evidence, combined with the safety data derived from clinical studies on the use of tezepelumab, provides concrete safety information.

## 7 Conclusion

The reported evidence suggests a correlation between the immunophlogosis that drives allergic diseases and the development of hematological neoplasms. The same cellular and molecular mechanisms that govern allergic inflammation can have a dual effect on immune surveillance mechanisms, positively or negatively influencing carcinogenesis. This duality of action can depend on various factors such as the tumor microenvironment, the organ affected by allergic inflammation, or the type of tumor. Although some mediators of allergic inflammation exhibit anti‐tumor activity, inhibiting these mechanisms through targeted therapies, such as monoclonal antibodies (mAbs), does not pose a risk of developing neoplasms. Our research demonstrate not only the safety of biological therapies with mAbs but also that the mechanisms underlying the anti‐tumor or pro‐tumor activity of Th2 inflammatory cells and cytokines still need in‐depth studies to be better understood. This evidence opens innovative avenues in hematologic‐oncology, potentially leading to the use of new diagnostic/prognostic biomarkers, which can also be used for patient stratification and the creation of prospective registries for biologic drugs. Further studies are needed to shed light on this topic and to facilitate significant advancements in the diagnosis, prevention, and treatment of hematologic‐oncological diseases.

Highlights:

— Th1/Th2 imbalance towards Th2‐related immune responses may play a crucial role in the progression and prognosis of hematological malignancies by creating an inflammatory environment that favors tumor cell proliferation.
— MCs involved in many allergic manifestations can influence tumor growth by releasing histamine and other inflammatory mediators that promote neoangiogenesis. A correlation between MC disorders and some types of hematological malignancies has been demonstrated.
— Eosinophilia can arise from malignant clones in myeloid malignancies or as a non‐neoplastic reactive process. CLL is the hematological malignancy most frequently associated with eosinophilic dermatitis (EDHM).
— ILCs can have pro‐ or anti‐tumor effects depending on the subset involved and the influence of many regulatory factors that can influence their activity.
— Alarmins play a dual role in promoting anti‐tumor immunity and tumor progression.
— Biological therapies, used in the treatment of severe allergic diseases and also in non‐purely allergic forms, do not seem to be able to affect tumor progression negatively.
— Although data on the association between the most recent biologicals and the incidence of hematological tumors are still limited, some of these could also have a therapeutic effect on some hematological tumors.

## Author Contributions

**Stefania Isola:** conceptualization, data curation, methodology, writing – original draft. **Luca Gammeri:** writing – original draft, methodology. **Federica Nuccio:** writing – original draft, methodology. **Alessandro Allegra:** writing – original draft. **Giorgio Walter Canonica:** writing – review and editing, supervision. **Sebastiano Gangemi:** supervision, writing – review and editing.

## Funding

The authors have nothing to report.

## Conflicts of Interest

Giorgio Walter Canonica reports research or clinical trials grants paid to his Institution from Menarini, AstraZeneca,GSK, Sanofi Genzyme and fees for lectures or advisory board participation from Menarini, AstraZeneca, CellTrion, Chiesi, Faes Farma, Firma, Genentech, Guidotti‐Malesci, GSK, HAL Allergy, Innovacaremd, Novartis, OM‐Pharma, Red Maple, Sanofi‐Aventis, Sanofi‐Genzyme, Stallergenes‐Greer and Uriach Pharma, outside the submitted work. The authors declare no conflicts of interest.

## Data Availability

The data that support the findings of this study are available from the corresponding author upon reasonable request.
